# Peroxiredoxin V (PrdxV) negatively regulates EGFR/Stat3-mediated fibrogenesis via a Cys48-dependent interaction between PrdxV and Stat3

**DOI:** 10.1038/s41598-019-45347-0

**Published:** 2019-06-19

**Authors:** Hoon-In Choi, Dong-Hyun Kim, Jung Sun Park, In Jin Kim, Chang Seong Kim, Eun Hui Bae, Seong Kwon Ma, Tae-Hoon Lee, Soo Wan Kim

**Affiliations:** 1 Department of Internal Medicine, Chonnam National University Medical School, Gwangju, Korea; 2 Department of Biochemistry, Dental Science Research Institute, School of Dentistry, Chonnam National University and Korea Mouse Phenotype Center, Gwangju, Korea

**Keywords:** Renal fibrosis, End-stage renal disease, Renal fibrosis, End-stage renal disease

## Abstract

Activation of the epidermal growth factor receptor (EGFR)/signal transducer and activator of transcription 3 (Stat3) signaling pathway has been reported to be associated with renal fibrosis. We have recently demonstrated that peroxiredoxin V (PrdxV) acted as an antifibrotic effector by inhibiting the activity of Stat3 in TGF-β-treated NRK49F cells. However, the underlying mechanism of PrdxV remains poorly understood. To investigate molecular mechanism of PrdxV, we used a transgenic mouse model expressing PrdxV siRNA (PrdxV^si^ mice) and performed unilateral ureteral obstruction (UUO) for 7 days. 209/MDCT cells were transiently transfected with HA-tagged WT PrdxV and C48S PrdxV. Transgenic PrdxV^si^ mice displayed an exacerbated epithelial-to-mesenchymal transition (EMT) as well as an increase in oxidative stress induced by UUO. In the UUO kidney of the PrdxV^si^ mouse, knockdown of PrdxV increased Tyr1068-specific EGFR and Stat3 phosphorylation, whereas overexpression of WT PrdxV in 209/MDCT cells showed the opposite results. Immunoprecipitation revealed the specific interaction between WT PrdxV and Stat3 in the absence or presence of TGF-β stimulation, whereas no PrdxV-EGFR or C48S PrdxV-Stat3 interactions were detected under any conditions. In conclusion, PrdxV is an antifibrotic effector that sustains renal physiology. Direct interaction between PrdxV and Stat3 through Cys48 is a major molecular mechanism.

## Introduction

Renal fibrosis is a principal process underlying the pathogenic progression of chronic kidney disease (CKD) and is characterized by excessive accumulation of extracellular matrix (ECM) in the interstitial compartment^1,2^. Increasing evidence indicates that activation of EGFR and the subsequent activation of its downstream intracellular pathways including extracellular signal-regulated kinases 1/2 (ERK1/2), phosphatidylinositol-3 kinase (PI3K)/AKT, and Stat3 contribute to the pathogenesis of renal fibrosis^3–6^. Blockage of EGFR-mediated downstream signaling pathways would have therapeutic potential for inhibiting the progression of renal fibrosis.

Peroxiredoxin V (PrdxV) is a thioredoxin peroxidase that reduces hydrogen peroxide, alkyl hydroperoxide, and peroxynitrite^7^. Its catalytic activity as a cytoprotective antioxidant is mediated by the formation of an intramolecular disulfide bond between the peroxidic (C_p_) cysteine 48 (Cys48) and the resolving (C_r_) Cys152 in the same polypeptide chain^8,9^. PrdxV has unique characteristics compared to those of other Prdx isotypes. PrdxV is robust to hyperoxidation by H_2_O_2_ ^10^ and exists in various subcellular locations such as the cytosol, nucleus, mitochondria, and peroxisomes^11^. Its expression is mainly regulated under pathophysiological conditions accompanied by an inflammatory response. For example, the expression of PrdxV is increased in response to innate immune signals, such as LPS/IFN-γ-treated mouse macrophages, LPS-treated microglia, and LPS-treated RAW264.7 cells, resulting in anti-inflammatory and antioxidant effects on host cells^12–15^. In renal pathophysiology research, proteomics data have been reported on renal protein networks regulated by knockdown of PrdxV after systemic hypoxia using PrdxV^si^ transgenic mice. In that study, PrdxV affected protein networks associated with oxidative stress, fatty acid metabolism, and mitochondrial dysfunction^16^. Recently, the expression of PrdxV was shown to be rapidly downregulated in the early fibrosis phase after UUO, and then ectopic expression of PrdxV in NRK49F cells functioned as an antifibrotic effector, inhibiting TGF-β-induced fibrosis by modulating Stat3 activation^17^. However, there is a lack of *in vivo* data studying transgenic mice engineered to have high or low expression levels of PrdxV.

The purpose of this study was to confirm the role of PrdxV as an antifibrotic effector and the molecular mechanism of PrdxV as a negative modulator of Stat3 *in vivo* using PrdxV^si^ transgenic mice. We observed that renal fibrosis induced by UUO was more severe in PrdxV^si^ mice than in PrdxV^wt^ mice and that this effect was associated with increased EGFR/Stat3 signaling pathway activity. Finally, we sought to elucidate the molecular mechanism underlying PrdxV and EGFR/Stat3 activation. We showed that PrdxV contributes to the negative regulation of TGF-β-induced fibrosis through the PrdxV-Stat3 interaction, which is dependent on the PrdxV catalytic cysteine.

## Results

### Histological correlation between renal fibrosis progression and PrdxV protein level after UUO

**Figure 1:**
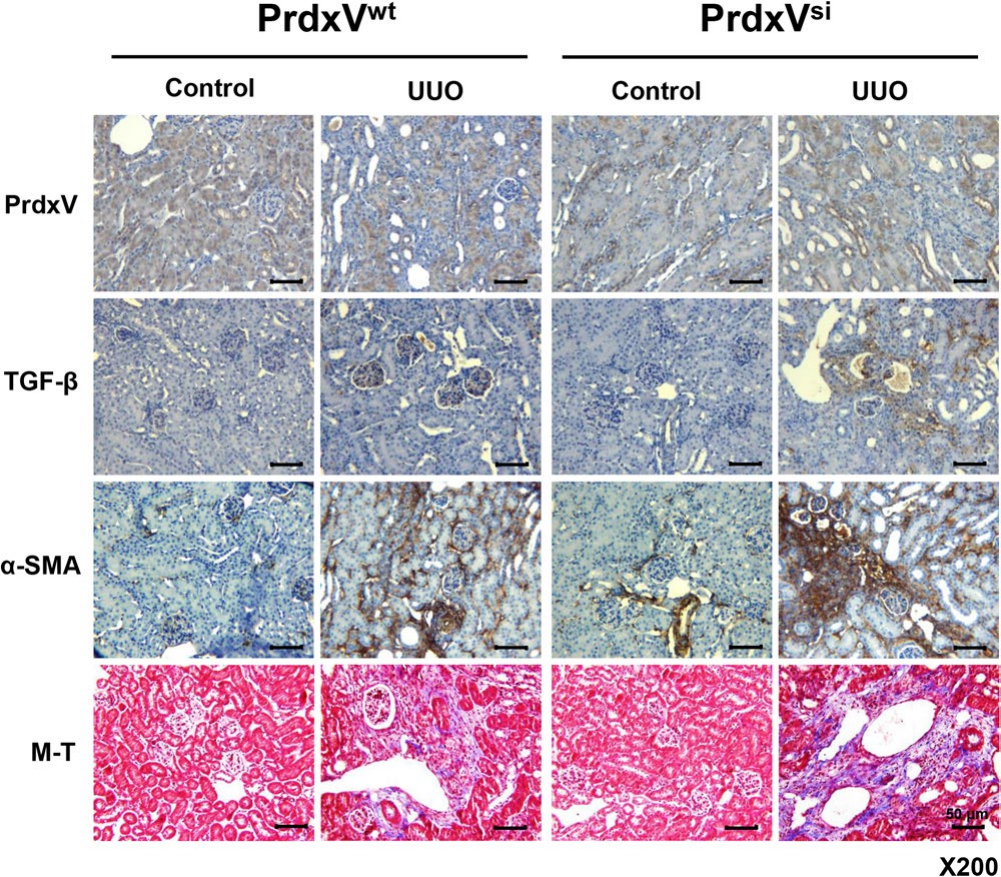
Immunohistochemistry (IHC) of fibrotic markers in UUO-induced PrdxV^wt^ and PrdxV^si^ mouse kidney. To compare the progression level of renal fibrosis by knockdown of PrdxV, we analyzed immunohistochemical expression of PrdxV and fibrotic marker proteins (TGF-β and α-SMA) as well as collagen deposition (blue color) by M-T staining of the renal cortex (Original magnification x200, Bar = 50 μm).

In our previous report^17^, we suggested a model for the physiological function and regulatory mechanism of PrdxV as an antifibrotic effector in TGF-β-treated NRK49F cells. To further determine the antifibrotic effect of PrdxV *in vivo*, we used PrdxV knockdown mice (PrdxV^si^ mice) that showed a reduction in the PrdxV protein level in the kidney^16^. First, we observed the expression of PrdxV in kidney of PrdxV^si^ mouse. The total amount of PrdxV protein in PrdxV^si^ mouse was reduced by about 40% compared to PrdxV^wt^ (Supplementary Fig. S1a). In immunohistochemistry, PrdxV was stained throughout cortex, outer medulla (OM), IM (inner medulla), and ISOM (inner strip of outer medulla) was stained most strongly. PrdxV^si^ mouse kidney was also found to be decreased histologically compared with PrdxV^wt^ mouse kidney (Supplementary Fig. S1b). PrdxV is also merged with the tubule-specific marker AQP1 (Proximal tubule and thin limbs of Henle), THP (thick ascending loop of Henle), Calbindin D28k (distal convoluted tubule and the connecting tubule), and AQP2 (collecting ducts and the connecting tubule), and its expression in each tubule was also reduced in the PrdxV^si^ mouse kidney (Supplementary Fig. S1c–f). Age-matched PrdxV^wt^ and PrdxV^si^ mice were subjected to UUO for 7 days. Progressive renal fibrosis was assessed by immunohistochemical staining for profibrotic markers, such as TGF-β and α-SMA, and Masson’s trichrome staining for collagen fibers of tissue sections from each group. As expected, the protein level of PrdxV was reduced in PrdxV^si^ kidney compared with that in PrdxV^wt^ kidney and was significantly reduced in the UUO group compared to that in the control group. On the other hand, the levels of TGF-β and α-SMA and accumulation of collagen fibers were more strongly detected in PrdxV^si^ mice than in PrxV^wt^ mice. We confirmed that the reduction in the PrdxV protein was related to an increase in UUO-induced renal fibrosis (Fig. 1).

### Knockdown of PrdxV promotes epithelial-to-mesenchymal transition (EMT)

**Figure 2:**
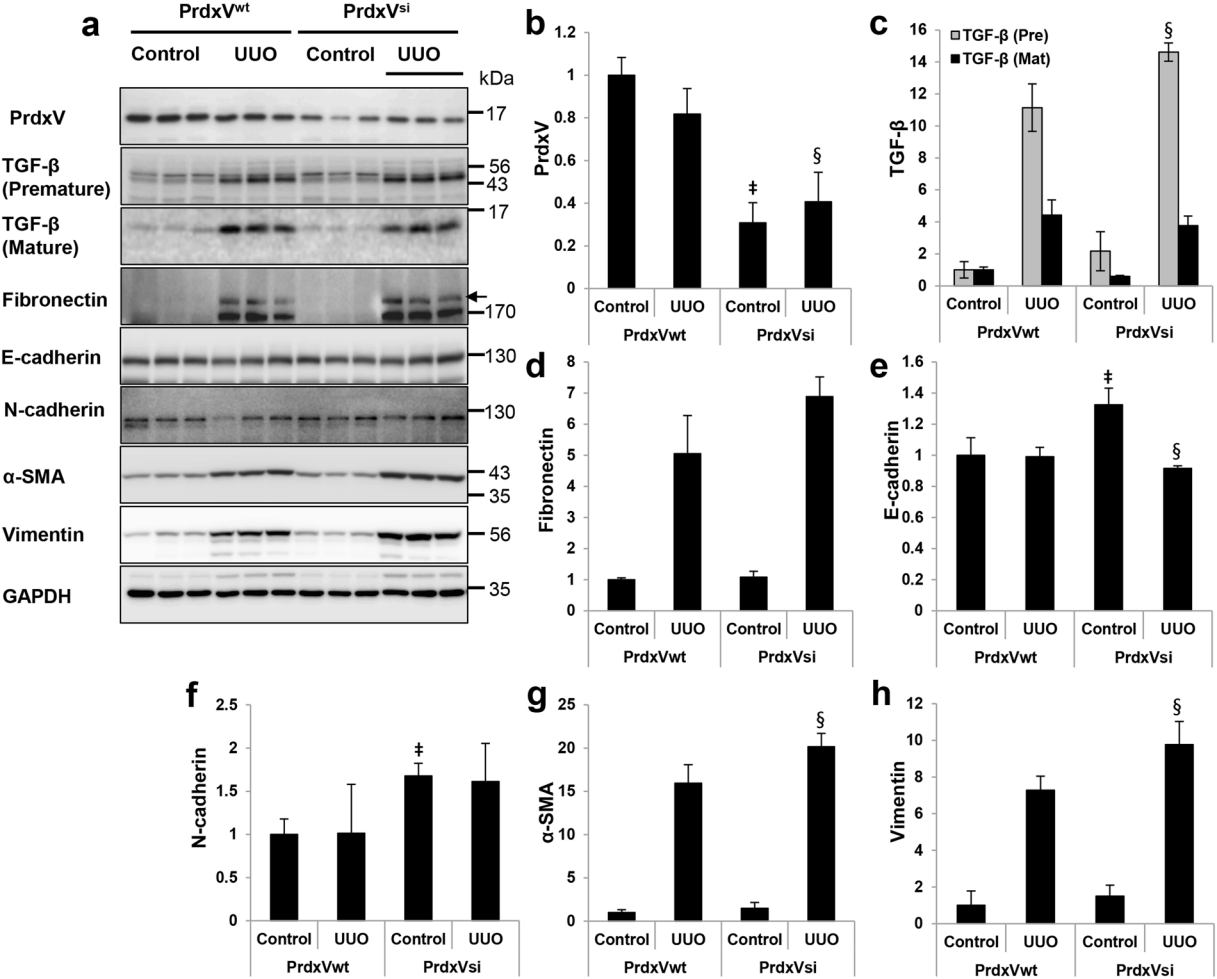
The protein expression of EMT markers in UUO-induced PrdxV^wt^ and PrdxV^si^ mouse kidney. **(a)** To confirm the increase in epithelial-to-mesenchymal transition by knockdown of PrdxV, whole kidney lysates were subjected to western blotting with specific antibodies against PrdxV and EMT markers. GAPDH was used as an internal control. **(b–h)** Bar graphs show the mean target protein/GAPDH expression as measured by densitometry (**b**; PrdxV, **c**; Premature & mature forms of TGF-β, **d**; Fibronectin, **e**; E-cadherin, **f**; N-cadherin, **g**; α-SMA, **h**; Vimentin). All values are presented as mean ± SD. Statistical significance was measured using the one-way ANOVA with the Fisher least significant difference post-test. ^‡^*p* < *0.05*; PrdxV^wt^ *vs*. PrdxV^si^ in control group, ^§^*p* < *0.05*; PrdxV^wt^ *vs*. PrdxV^si^ in UUO group.

EMT of tubular epithelial cells is a characteristic of renal fibrosis^18^. Therefore, we examined the expression patterns of EMT markers in order to confirm the occurrence of renal fibrosis caused by differences in protein expression of PrdxV between PrdxV^wt^ and PrdxV^si^ mice. The total protein level of PrdxV was reduced in PrdxV^si^ mouse kidney. Expression of E-cadherin, a marker of epithelial cells, in the UUO kidney of PrdxV^si^ mice was reduced to a greater extent than that in PrdxV^wt^ mice. However, the expression of fibronectin, N-cadherin, α-SMA and vimentin, which are characteristic of mesenchymal cells, showed a significant increase or a tendency to increase in the UUO kidney of PrdxV^si^ mice (Fig. 2). These results suggest that PrdxV^si^ mice are more vulnerable to UUO-induced renal fibrosis than PrdxV^wt^ mice.

### Knockdown of PrdxV increases UUO-induced oxidative stress

**Figure 3:**
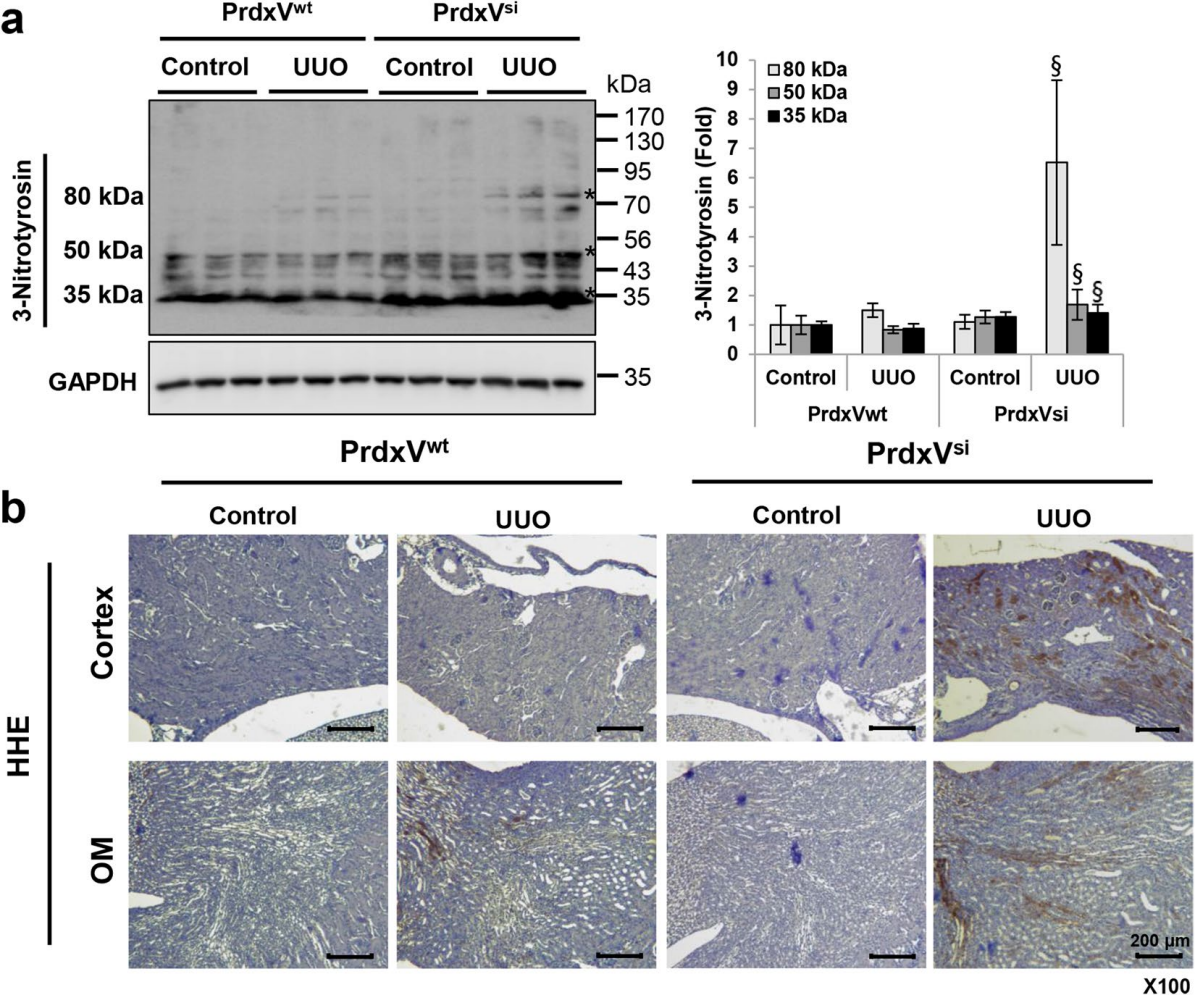
The level of oxidative stress markers in UUO-induced PrdxV^wt^ and PrdxV^si^ mouse kidney. As one of the major mechanisms of renal fibrosis induced by UUO, the level of oxidative stress was assessed in both PrdxV^wt^ and PrdxV^si^ mouse kidneys. **(a)** The increased nitrotyrosine level in UUO-induced PrdxV^si^ kidney. The content of nitrotyrosine, as a protein oxidative marker, was analyzed by western blotting conducted with whole kidney lysates. Three major bands of different sizes were detected at 80 kDa, 50 kDa, and 35 kDa. GAPDH was used as an internal control. **(b)** The increased HHE level in UUO-induced PrdxV^si^ kidney. The content of HHE, as a lipid peroxidation marker, was analyzed by immunohistochemistry with a specific anti-HHE antibody. Image was magnified at x100, Bar = 200 μm. All values are presented as mean ± SD. Statistical significance was measured using the one-way ANOVA with the Fisher least significant difference post-test. ^§^*p* < *0.05*; PrdxV^wt^ *vs*. PrdxV^si^ in UUO group.

Oxidative stress, characterized by increases in reactive oxygen species (ROS) and/or reactive nitrogen species (RNS), is also one of the pathogenic factors leading to renal diseases^19,20^. In particular, PrdxV among peroxiredoxin isotypes is a peroxynitrite reductase with a high rate constant for peroxinitrite, (7 ± 3) × 10^7^ M^−1^ s^−1^ ^21^. Recently, it has been reported that PrdxV acts as a negative feedback loop suppressing NO production^22^. Thus, we wondered whether there was an association with oxidative stress in promoting the progression of UUO-induced renal fibrosis in the kidney of PrdxV^si^ mice. We indirectly examined oxidatively damaged protein products, such as the modified amino acid 3-nitrotyrosine (3-NT), via western blotting and the extent of hydroxyhexanal (HHE), a lipid peroxidation marker, through immunohistochemistry (IHC). Three oxidative protein bands modified by 3-NT were detected at 80 kDa, 50 kDa and 35 kDa, and the band densities were increased in the UUO kidney of PrdxV^si^ mice compared with those of PrdxV^wt^ mice (Fig. 3a). Similarly, we observed that the HHE content also showed significantly greater distribution in the kidney tissue of the UUO group of PrdxV^si^ mice than in that of PrdxV^wt^ mice. These results indicate that the knockdown of PrdxV is strongly associated with UUO-induced oxidative stress (Fig. 3b).

### Downregulation of PrdxV activates the EGFR/Stat3 signaling pathway

**Figure 4:**
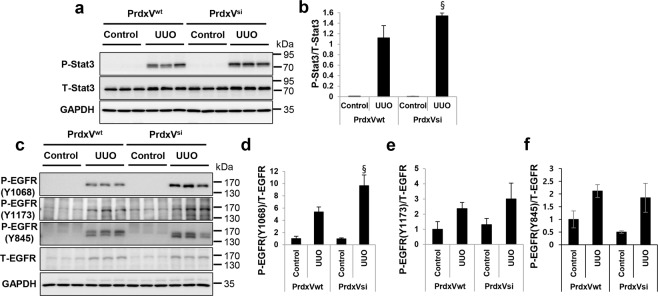
Activation of EGFR and Stat3 in UUO-induced PrdxV^si^ kidney. To further verify the involvement of the EGFR and Stat3 signaling pathway in renal fibrosis aggravated by knockdown of PrdxV, the expression levels and activation levels of Stat3 and EGFR as an upstream molecule of Stat3 activation were assessed by western blotting. **(a,b)** Stat3 activation. Stat3 activation was analyzed by measuring phosphorylation at Tyr705 in Stat3. **(c–f)** Site-specific EGFR phosphorylation. The phosphorylation of EGFR at Tyr1068 was assessed with a specific anti-pEGFR Tyr1068 antibody. The phosphorylation levels of EGFR at Tyr1173 and Tyr845 were also checked as negative controls. Bar graphs show the mean ratios of the phosphorylated forms to the total level of the indicated targets as measured by densitometry. GAPDH was used as an internal control. All values are presented as mean ± SD. Statistical significance was measured using the one-way ANOVA with the Fisher least significant difference post-test. ^§^*p* < *0.05*; PrdxV^wt^ *vs*. PrdxV^si^ in UUO group.

Next, we asked which signaling pathway resulted in promoting renal fibrosis due to knockdown of PrdxV. To answer this question, we examined the activation of the signaling pathway that plays an important role in renal fibrosis. In our previous report, we mentioned that PrdxV overexpression negatively modulates TGF-β-induced Stat3 activation, even though an upstream molecule involved in the activation of Stat3 has not been found^17^. We therefore wanted to reaffirm whether, among the many noncanonical TGF-β signaling pathways, activation of Stat3 by knockdown of PrdxV is one of the signaling pathways of renal fibrosis induced by UUO, and if so, whether any upstream molecules are involved in the activation of Stat3. Consistent with the *in vitro* data, knockdown of PrdxV promoted the activation of Stat3 rather than the activation of Smad2/3 by UUO (Fig. 4a,b and Supplementary Fig. S2). Interestingly, site-specific phosphorylation at Tyr1068 of EGFR, which is known to be associated with the activation of Stat3^23^, was higher in the UUO group of PrdxV^si^ mice than that in PrdxV^wt^ mice. There was no difference between the UUO-induced PrdxV^wt^ and PrdxV^si^ groups with regard to phosphorylation of EGFR at Tyr1173 and Tyr845 (Fig. 4c–f). These results suggest that activation of Stat3 by the activation of site-specific EGFR at Tyr1068 is one of the possible mechanisms for promoting renal fibrosis in UUO-induced PrdxV^si^ mice.

### Upregulation of PrdxV negatively modulates the activation of site-specific EGFR (Tyr1068) and Stat3

**Figure 5:**
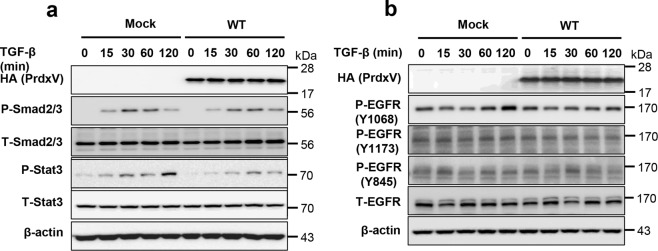
Negative regulation of EGFR and Stat3 by PrdxV in TGF-β treated 209/MDCT cells. To confirm the regulation of the EGFR/Stat3 axis by PrdxV, HA-tagged WT PrdxV and Mock were transiently expressed in 209/MDCT cells and treated with TGF-β (10 ng/ml) for the indicated times (0, 15, 30, 60, and 120 min). Activation of the TGF-β-mediated signal transducer was compared with site-specific phosphorylation levels of each signaling molecule. **(a)** Smad2/3 and Stat3 activation. Smad2/3 activation was analyzed by the phosphorylation level at Ser465/467 in Smad2 and Ser423/425 in Smad3. In addition, Stat3 activation was analyzed by the phosphorylation level at Tyr705. **(b)** EGFR activation. EGFR activation was analyzed by site-specific phosphorylation levels at Tyr845, Tyr1068, and Tyr1173. The total protein level of each signaling molecule was also checked in both groups. GAPDH were used as an internal control.

*In vivo* experiments suggested the activation of site-specific EGFR (Tyr1068) and subsequent activation of Stat3 as a mechanism for progressive renal fibrosis in UUO-induced PrdxV^si^ mice. Therefore, to confirm this mechanism, we reaffirmed the relationship between PrdxV and the EGFR/Stat3 signaling pathway by overexpressing the HA-tagged mouse wild-type PrdxV (WT) in 209/MDCT cells, a mouse distal convoluted tubule cell line. Consistently, overexpression of WT PrdxV in 209/MDCT cells inhibited the activity of Stat3 by TGF-β treatment compared to that in Mock but did not significantly alter the activity of Smad2/3 (Fig. 5a). In addition, compared to that in Mock, overexpression of WT PrdxV in 209/MDCT cells inhibited Tyr1068-specific phosphorylation of EGFR, one of the upstream molecules of Stat3. There was no difference between the TGF-β-treated Mock and WT PrdxV with regard to the phosphorylation of EGFR at Tyr1173 and Tyr845 (Fig. 5b). These results imply that PrdxV negatively regulates EGFR (Tyr1068)-mediated Stat3 activation.

### PrdxV regulates vimentin activation in a peroxidatic cysteine (Cys48)-dependent manner

**Figure 6:**
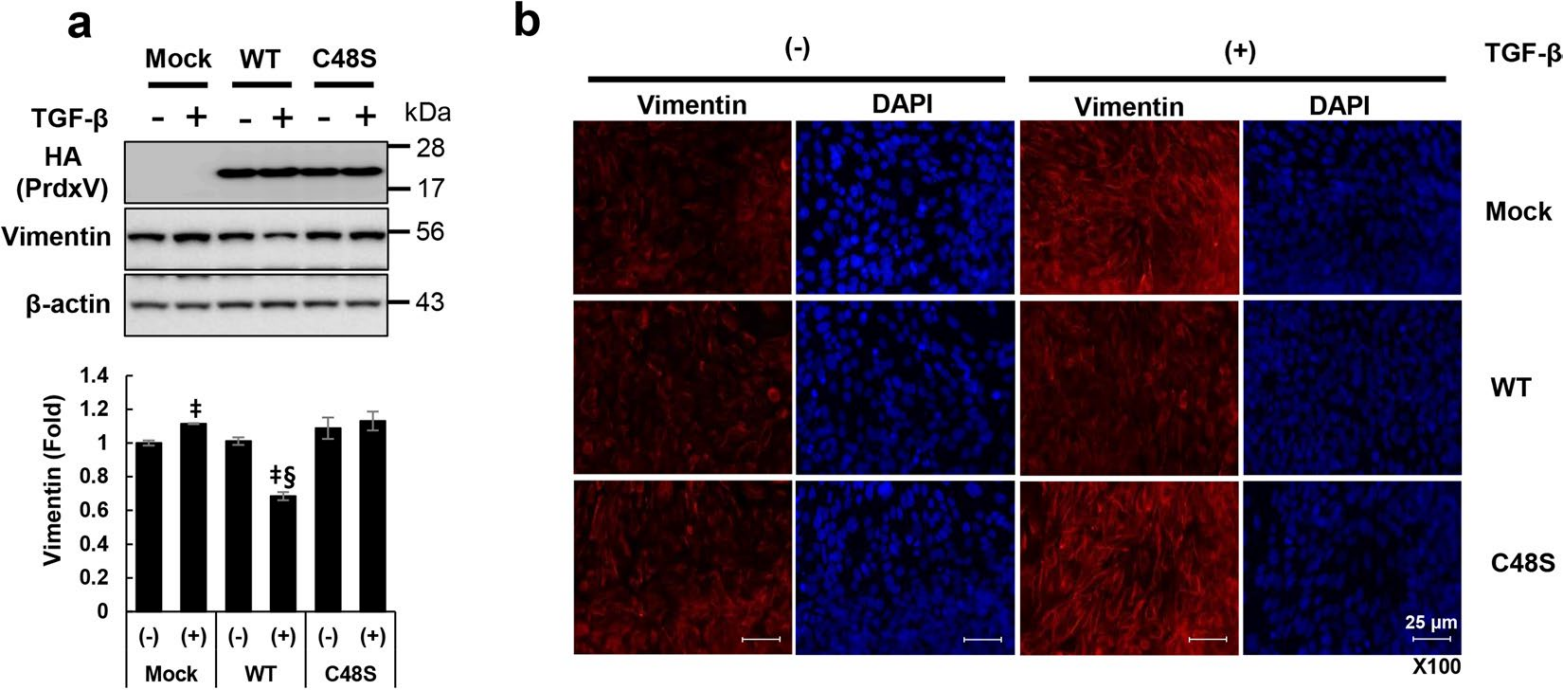
Catalytic cysteine (Cys48)-dependent modulation of vimentin by PrdxV. Vimentin is a characteristic marker of mesenchymal cells, and its promoter includes a Stat3 binding site. Therefore, to assess the regulation of vimentin, as a direct target of Stat3, by PrdxV, HA-tagged WT PrdxV and Cys48Ser PrdxV constructs were transiently expressed in 209/MDCT cells. Then, in the absence or presence of TGF-β, the protein level of vimentin was assessed with **(a)** western blotting and **(b)** immunofluorescence (IF) staining. Bar graphs show the mean protein expression of vimentin/β-actin as measured by densitometry. All values are presented as mean ± SD. Statistical significance was measured using the one-way ANOVA with the Fisher least significant difference post-test. ^‡^*p* < *0.05*; TGF-β treated vs. untreated in each group. ^§^*p* < *0.05*; TGF-β-treated Mock vs. WT or Cys48Ser. Image was magnified at x200, Bar = 25 μm.

Activation of Stat3 induces the expression of multiple genes that results in the progression of renal fibrosis. In particular, among several fibrotic markers, it is known that Stat3 directly binds to the promoters of some fibrotic markers such as Twist, Zeb1, and vimentin to regulate their gene expression^24^. Interestingly, upregulation of vimentin in TGF-β-treated 209/MDCT cells was significantly reduced by WT PrdxV overexpression compared to Mock. We also observed that negative regulation of vimentin by overexpression of WT PrdxV was not found in the Cys48Ser PrdxV mutant with catalytic Cys48 substituted with serine treated with TGF-β (Fig. 6a). Consistent with immunoblotting, the immunofluorescence data also confirmed that the increase in vimentin, which is characteristic of mesenchymal cells treated with TGF-β, was reduced by WT PrdxV but was still increased by Cys48Ser PrdxV (Fig. 6b) in TGF-β-treated 209/MDCT cells. These results reaffirmed that PrdxV has an antifibrotic effect and suggested that there is a mechanism dependent on catalytic Cys48.

### PrdxV interacts with Stat3 via catalytic Cys48

**Figure 7:**
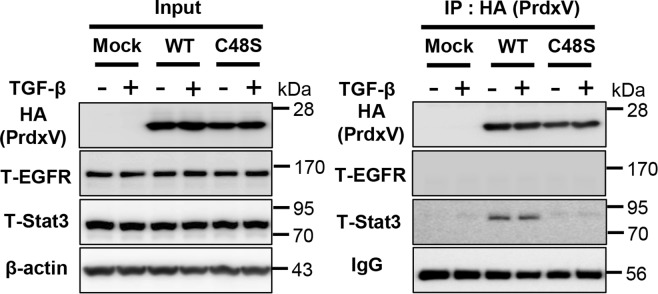
Direct interaction between PrdxV-Stat3 via Cys48. To understand the negative regulatory mechanism of PrdxV in EGFR/Stat3-mediated fibrosis, we tried to confirm the protein-protein interaction between these molecules. HA-tagged WT PrdxV, C48S PrdxV, and Mock were transiently expressed in 209/MDCT cells. After TGF-β treatment for 2 hours, cells were harvested, and immunoprecipitation was performed using HA-agarose as described in the Materials & Methods section. The immunoprecipitated sample was separated by SDS-PAGE (reducing gel) and immunoblotted using anti-HA, anti-total EGFR, and anti-total Stat3 antibodies. Heavy chain IgG was used as a loading control (right panel). Expression of each protein used for immunoprecipitation was also determined using the same antibodies. β-actin served as a loading control (left panel).

Next, we sought to determine which molecular mechanism of PrdxV has an antifibrotic effect based on the following results we obtained: The first showed that the EGFR/Stat3 pathway is the primary signal, and the second showed that the mechanism works in a Cys48-dependent manner. We therefore overexpressed HA tagged-Mock, WT PrdxV, and C48S PrdxV in 209/MDCT cells and observed the presence of protein-protein interactions between EGFR/Stat3 signaling molecules and PrdxV induced by TGF-β treatment. From our data, we expected an interaction between PrdxV and EGFR because PrdxV negatively regulated EGFR (Tyr1068)-mediated Stat3 activation, but interestingly, a specific interaction between WT PrdxV and Stat3 was detected in the absence or presence of TGF-β stimulation, whereas no PrdxV-EGFR or C48S PrdxV-Stat3 interactions were detected under any conditions (Fig. 7). Given these findings, we propose that PrdxV specifically and directly interacts with Stat3 via Cys48 to regulate renal fibrosis. However, further studies on the molecular linkage mechanism by which EGFR Tyr1068 mediates the regulation of Stat3 by PrdxV as well as the intermolecular disulfide bond between PrdxV and Stat3 will be needed.

## Discussion

**Figure 8:**
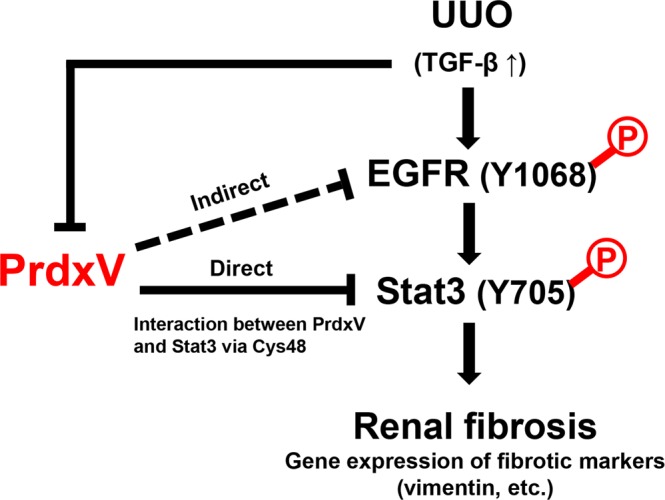
Proposed model for antifibrotic effect of PrdxV. Under the pathological conditions such as UUO, UUO kidney increases the expression of TGF-β, a fibrotic cytokine, which promotes the activation of EGFR/Stat3, one of the noncanonical TGF-β signal pathways. In particular, the activation of Tyr1068 of EGFR serves as a docking site for the phosphorylation of Tyr705 of Stat3. Activated dimeric Stat3 increases the expression of fibrotic markers such as vimentin, which is regulated by stat3 in the nucleus, leading to renal fibrosis. In control kidney under normal physiological conditions, PrdxV binds to Stat3 in a catalytic Cys48-dependent manner and its binding plays a role in inhibiting the activation of EGFR/Stat3. Therefore, a decrease in PrdxV under fibrotic stress has an adverse effect on the maintenance of PrdxV-Stat3 interaction and leads to activation of the EGFR/Stat3 pathway.

Consistent with our previous *in vitro* data^17^, we have confirmed *in vivo* that the physiological function of PrdxV is protective against renal fibrosis (Fig. 8). PrdxV expression was inversely related to the progressive process of renal fibrosis. In fact, the UUO kidney of PrdxV^si^ mice showed an increase in the immunohistochemical or protein expression of fibrotic markers compared to that of PrdxV^wt^ mice. Recent studies have reported that the expression of PrdxV is regulated by the activity and expression of dimethylarginine dimethylaminohydrolase (DDAH). DDAH is involved in the degradation of the asymmetric dimethylarginine (ADMA), an endogenous inhibitor of nitric oxide synthase (NOS), and plasma ADMA accumulation and DDAH1 activity/expression reduction are linked to CKD pathology^25^. In Ddah1−/− tubular epithelial cells (TECs), a deficiency of DDAH is accompanied by a subsequent protein reduction in PrdxV, which leads to EMT of TECs in an AMPK-mediated manner^26^. In this study, we clarified that PrdxV levels were reduced in fibrotic kidneys, a pathological feature of CKD, and PrdxV in renal physiology functions as an antifibrotic effector.

In pathological conditions, the generation of free radicals and/or depletion of the antioxidant defense system leads to higher levels of ROS/RNS, resulting in tissue damage. The kidney is an organ highly vulnerable to damage caused by oxidative stress, likely due to the abundance of long-chain polyunsaturated fatty acids (such as PUFAn-6) in the composition of renal lipids^27,28^. In agreement with this, we observed that the UUO kidney of PrdxV^si^ mice was strongly stained with 4-HHE, an aldehyde product of lipid peroxidation of PUFAn-6, as well as 3-NT, a protein marker of oxidative stress. Indeed, as reported in many studies, there is no doubt that ROS/RNS generation is increased in tissues from patients with diabetes and obesity^29,30^.

Stat3 is a transcription factor that mediates the intracellular signaling induced by various cytokines, hormones, and growth factors^31^. Recent studies suggested that Stat3 is a central regulator of the molecular link between tubular and interstitial cells during CKD progression^32^, and pharmacologic inhibition of Stat3 has been shown to decrease fibrotic progression in diabetes or UUO^4,33^. Consistent with previous results in our TGFβ-treated NRK49F cells^17^, our results confirmed that renal fibrosis in PrdxV^si^ mice was increased by the activation of Stat3 rather than the activation of Smad2/3. In addition, we also observed that site-specific phosphorylation of EGFR at Tyr1068 was activated by Stat3 phosphorylation in the UUO kidney of PrdxV^si^ mice. We also observed that the overexpression of WT PrdxV in 209/MDCT cells inhibited the activity of EGFR (Tyr1068) as well as that of Stat3. Persistent activation of EGFR signaling is critically associated with the development and progression of renal fibrosis^3^. In Ang II-infused or UUO models, either genetic (dominant negative overexpression and proximal tubule-specific null mouse) or pharmacologic inhibition (erlotinib and gefitinib) of EGFR induced significantly less tubulointerstitial injury in the kidney than was observed in the wild type^6,34–36^. This inhibitory effect was the mechanism by which ERK1/2 activity was inhibited by Src-mediated EGFR Tyr845 phosphorylation, starting with ROS generation (ROS-Src-EGFR-ERK signaling)^34^. EGFR is phosphorylated at various site-specific tyrosine residues that are involved in different cellular responses^23^. In this study, it was observed that PrdxV specifically acts on the phosphorylation at Tyr1068 rather than at Tyr845 or Tyr1173 to regulate EGFR activity. EGFR signaling can be transferred to Stat3^37^. Stat3 interacts with EGFR at Tyr1068 or Tyr1086 sites in the cytoplasmic domain, and it is activated by phosphorylation at Tyr705^38–40^. Given these results, we can suggest that PrdxV functions as a negative modulator of the EGFR/Stat3 axis.

Classically, activation of Stat3 is initiated by phosphorylation at Tyr705 of cytoplasmic Stat3 after stimulation. Then, phosphorylated Stat3 is homodimerized and transported to the nucleus where it regulates the expression of the target gene^41^. Activated Stat3 triggers the expression of potential paracrine targets involved in tubulointerstitial communication, such as Lcn2, Pdgfb, and Timp1^32^. In addition, Stat3 is a DNA-binding transcription factor that directly regulates a variety of fibrotic target genes with a Stat3 binding site in the promoter, such as Twist, Zeb1, and vimentin^24^. Our data also showed that the upregulation of TGF-β-treated vimentin was inhibited by overexpression of WT PrdxV, but not C48S PrdxV, in 209/MDCT cells. The antifibrotic effect of PrdxV could be explained by the Stat3- and catalytic Cys48-dependent manner in which the underlying mechanism functions.

Recently, it has been reported that H_2_O_2_-mediated Stat3 signaling is regulated by the formation of disulfide exchange intermediates between Prdx2 and Stat3^42^. In the peroxidase-mediated redox signaling model, Prdx2 acts as a receptor that senses the H_2_O_2_ signal and transfers this oxidative equivalent to the redox regulated transcription factor Stat3, taking the form of thiol-disulfide exchange intermediates, and the transcriptional activity of Stat3 rapidly and specifically, and transiently controlled. In this study, it is interesting that PrdxV directly interacts with Stat3 to negatively regulate the EGFR/Stat3 axis, even though EGFR (Tyr1068) is an upstream molecule of Stat3 activity. In addition, the direct interaction between PrdxV and Stat3 is dependent on catalytic Cys48. However, even if Cys48 of PrdxV plays an important role in interaction with Stat3, further studies are needed to determine whether the interaction of PrdxV and Stat3 is due to intermolecular disulfide bonds. As in the case of Prdx2-mediated Stat3 regulation, further studies are needed to determine whether PrdxV modulates renal fibrosis by peroxidase-mediated redox signaling in regulating TGF-B-induced Stat3 activation. In the EGFR-Stat3 interaction, Tyr1068 and Tyr1086, containing the YXXQ motif, are Stat3-preferential binding sequences in EGFR^43^. Therefore, further research is needed on how PrdxV modulates the EGFR-Stat3 interaction through the PrdxV-Stat3 interaction.

In conclusion, *in vivo*, our findings show a critical role for the inhibitory effect of PrdxV in EGFR/Stat3 activation associated with the development of renal fibrosis after UUO. In particular, it suggested that the molecular mechanism, as a negative modulator, is due to the Cys48-dependent interaction between PrdxV and Stat3. PrdxV may be a candidate target for renal fibrosis treatment in CKD patients.

## Materials and Methods

### Animals

The animal experiments were approved by the Animal Care Regulations (ACR) Committee of Chonnam National University Medical School (CNU IACUC-H-2016-49) and our protocols conformed to the institution guidelines for experimental animal care and use. Experiments were performed using male PrdxV knock-down mice (PrdxV^si^ mice)^16^ and wild type littermate mice (PrdxV^wt^ mice) as a control (8–12 weeks old). Mice were housed under controlled temperature (21 ± 2 °C) in a 12 h light-dark cycle.

### Reagents and antibodies

Recombinant transforming growth factor beta 1 (TGF-β1) was purchased from Peprotech (Cat# 100-21, Korea). Anti-HA (hemagglutinina) agarose gel was from Sigma-aldrich (Cat# E6779, St. Louis, MO). Antibodies against phospho-Stat3 (Tyr705), total-Stat3, total-Smad2/Smad3, phospho-Smad2 (Ser465/467)/Smad3 (Ser423/425), TGF-β, N-cadherin, vimentin, HA tag, Smad4, nitrotyrosine, Total-EGFR, phospho-EGFR (Y845, Y1068, Y1173), total-Src, and phospho-Src (Y416) were all from Cell Signaling Technology (Danvers, MA). TGFβRI, TGFβRI, AQP1 and Smad7 was from Santa Cruz (Dallas, Texas). E-cadherin and fibronectin were from BD Biosciences (Franklin Lakes, NJ). AQP2 was from Novus Biologicals (Littleton, CO, USA). THP was from AbD serotec (Kidlington, United Kingdom). Antibodies against α-SMA, calbindin D28K and β-actin were from Sigma-Aldrich (St. Louis, MO). Specific antibody against PrdxV was gifts from Dr. Ho Zoon Chae (Chonnam National University, Korea).

### Unilateral ureteral obstruction (UUO) animal model

Both PrdxV^wt^ mice and PrdxV^si^ mice were divided to two groups (Control vs UUO; n = 8, each group). Unilateral ureteral obstruction was induced by ligation of the left ureter for seven days. The abdominal cavity was opened, and 2–0 silk ligature was placed at left proximal ureter under anesthesia with ketamine (50 mg/kg, intraperitoneally; Yuhan, Seoul, Korea). The control group received the same treatment, with the exception of the ligature.

### Cell culture and TGF-β treatment

209/MDCT, a mouse distal convoluted tubule cells (ATCC, Manassas, VA), were cultured in a 1:1 ratio mixture of DMEM with 1 g/L glucose and 1 mM sodium pyruvate (Life Technologies, Cat#11885) and Ham’s F-12 Nutrient Mix, (Life Technologies Cat# 11765). Complete medium was supplement with 5% FBS, 50 U/mL penicillin and 50 μg/mL streptomycin at 37 °C under a humidified 5% CO_2_ atmosphere. For transient expression of PrdxV in 209/MDCT cells, HA-tagged mouse wild type (WT) PrdxV and cysteine mutant (C48S) PrdxV substituted Cys48 in active site cysteine of PrdxV to Ser, were used^14^. Plasmid DNA constructs were transfected into 209/MDCT cells using Fugene HD transfection reagent (Promega, Madison, WI) by 1:3 ratio of DNA to transfection reagent. After 1 day transfection, the cells were starved with serum free media for another one day, followed by TGF-β (10 ng/ml) treatment for the indicated times.

### Immunohistochemistry (IHC) and Masson’s trichrome (M-T) staining

Kidney specimens were fixed in 10% formalin then immersed in phosphate buffer saline (PBS). The tissues were embedded in paraffin and cut into 4-μm sections. Standard immunohistochemical (IHC) protocol was also followed after deparaffinization and rehydration. The specimens were incubated with the primary antibodies: rabbit polyclonal antibodies directed against PrdxV (diluted 1:1000; made by Prof Ho Zoon Chae, Chonnam National University, Republic of Korea), rabbit polyclonal antibodies directed against TGF-β (diluted 1:200; Cell Signaling Technology, Danvers, MA, USA), and mouse monoclonal antibodies directed against α-SMA (diluted 1/1000; Sigma Chemical Co., St. Louis, MO) at 4 °C overnight. For detection of lipid peroxidation products in kidney tissue, the specimens were incubated with mouse anti-HHE (4-hydroxy-2-hexenal) antibody (diluted 1/50; COSMO BIO., LTD, Tokyo, Japan), and then, tissue sections were incubated with HRP-conjugated secondary antibody (diluted 1/50; Cell Signaling Technology, Danvers, MA, USA) for 1 hour at room temperature. Signals were developed with diaminobenzidine (DAB) chromogenic substrate (DakoCytomation) and counterstained with hematoxylin. The stained images were examined under three randomly selected fields (×200; for PrdxV, TGF-β, and α-SMA) and (×100; for HHE). For M-T staining, Masson’s tricolor staining kit (Polysciences, Inc., Warrington, Pa., USA) was used according to commercial protocols. Interstitial fibrosis was assessed at 200× magnification on Masson’s trichrome‐stained sections using three randomly selected fields for each animal.

### Immunocytochemistry (ICC)

209/MCDT cells were seed onto four well-cell culture slides (2 × 10^4^/well) and were proceed as mentioned previous. Cells were washed with PBS and were fixed in 4% paraformaldehyde for 10 min. Subsequently, cells were permeablized with permeabilization buffer (0.5% Triton X-100 in PBS) and the slides were incubated with primary antibodies to vimentin (Cat# 5741, 1/200 dilution) in diluted with equilibration buffer (1% BSA, 0.5% Triton X-100 in PBS) at 4 °C overnight. Following incubation with primary antibody, the cells were washed with equilibration buffer and incubated for 1 h at room temperature with anti-Rabbit Cy3- conjugated secondary antibodies (Abcam). The nuclei were counterstained using SlowFade Gold antifade reagent with DAPI (Invitrogen). Images were captured using a confocal microscope (LSM 510; Carl Zeiss). Image was magnified at x200, Bar = 25 μm.

### Immunoprecipitation assay

One day prior to transfection, 209/MDCT cells were seed into 100 mm dishes at 2.5 × 105 cell per dish. The cells then transfected with HA tagged PrdxV (WT or C48S; 2 μg). Twenty-four hours after transfection, the cells were incubated for 2 h in the absence or presence of 10 ng/ml of TGF-β and then lysed in 900 μl of cold lysis buffer (20 mM Tris-HCl, pH 7.5, 100 mM NaCl, 1 mM EDTA, 0.5% Triton X-100, 1 mM NaF, 1 mM Na_3_VO_4_, 0.1 mM AEBSF, 1 μg/ml aprotinin, 0.5 μg/ml leupeptin, 1 mM PMSF). After centrifugation, samples of the supernatants (1 mg) were separately immunoprecipitated using HA-agarose beads (prewashed 10 μl of a 50% slurry) for 12 h at 4 °C. The beads were then washed three times with cold PBS, and the bound protein was recovered by boiling in SDS-PAGE sample buffer, separated by SDS-PAGE on a 13.5% gel or 8% gel, and immunoblotted with indicated antibodies. In a separate protocol, the expression level of each protein used for immunoprecipitation was also determined using the same antibodies.

### Statistics

Values are presented as mean ± standard deviation (S.D.). Between-group differences were measured using one-way ANOVA with Fisher least significant difference post hoc analysis where appropriate. P-values < 0.05 were considered as statistically significant. All experiments were performed at least three times.

## Supplementary information

Supplementary information

## Data Availability

Uncropped western blotting data supporting this article have been uploaded as part of the Supplementary Information.
