# Supplementary material for: Peroxiredoxin V (PrdxV) negatively regulates EGFR/Stat3-mediated fibrogenesis via a Cys48-dependent interaction between PrdxV and Stat3
Source: Sci Rep. 2019 Jun 19;9:8751. doi: 10.1038/s41598-019-45347-0 (PMC6584630; doi:10.1038/s41598-019-45347-0)

Article

Peroxiredoxin V (PrdxV) negatively regulates EGFR/Stat3-mediated fibrogenesis via a Cys48-dependent interaction between PrdxV and Stat3

Hoon-In Choi[1], Dong-Hyun Kim[1], Jung Sun Park[1], In Jin Kim[1], Chang Seong Kim[1], Eun Hui Bae[1], Seong Kwon Ma[1], Tae-Hoon Lee[2], and Soo Wan Kim[1*]

[1]Department of Internal Medicine, Chonnam National University Medical School, Gwangju, Korea and [2]Department of Biochemistry, Dental Science Research Institute, School of Dentistry, Chonnam National University and Korea Mouse Phenotype Center, Gwangju, Korea

*Corresponding Author

Full name: Dr. Soo Wan Kim

Department: Department of Internal Medicine

University/Hospital: Chonnam National University Medical School

Street Name & Number: 42 Jebongro

City, State, Postal code, Country: Gwangju, 61469, Korea

Tel: +82 62 220 6271

Fax: +82 62 225 8578

E-mail: skimw@chonnam.ac.kr

Supplementary Figures.

Supplementary Figure. S1

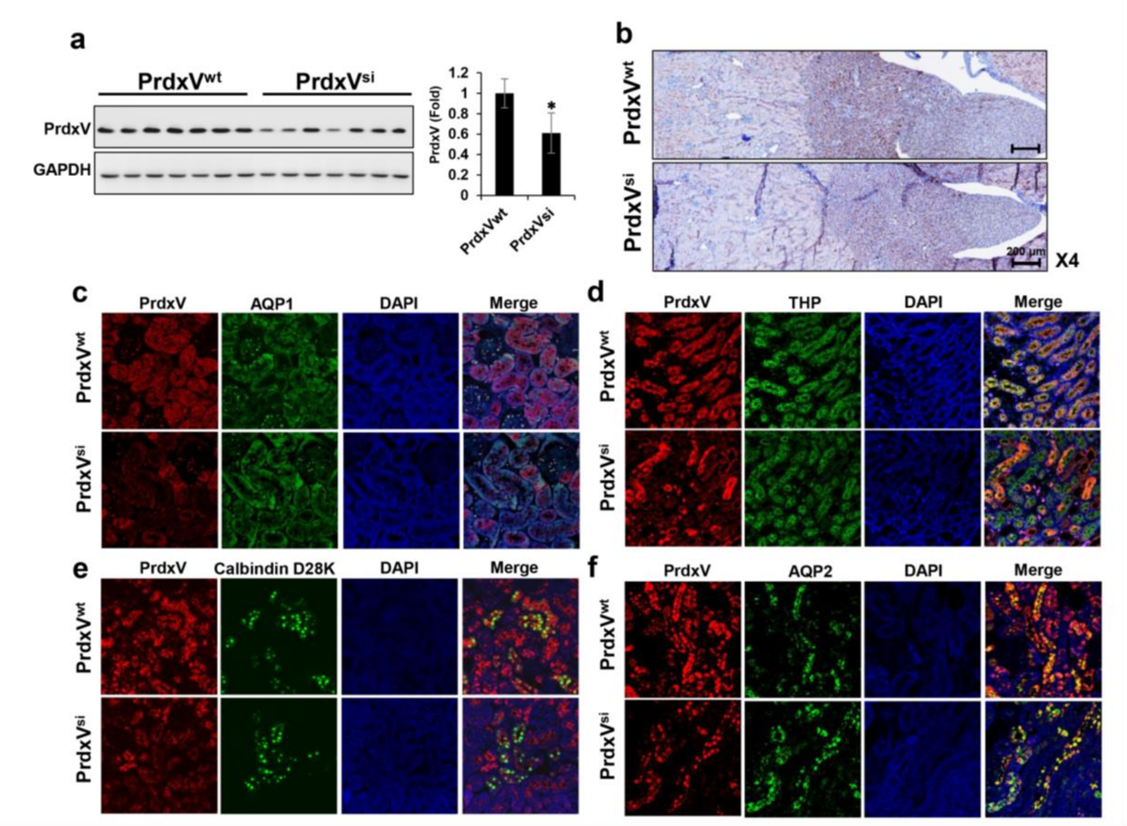

Comparison of PrdxV expression between PrdxV^{wt} and PrdxV^{si} mouse kidney. **(a)** Total protein level of PrdxV were assessed by western blotting. GAPDH was used as an internal control. Bar graphs show the mean PrdxV protein/GAPDH expression as measured by densitometry. $*p < 0.05$; PrdxV^{wt} *vs.* PrdxV^{si}. **(b)** Light micrographs illustrating immunostaining of PrdxV of PrdxV^{wt} and PrdxV^{si} kidney. Immunolabeling is present from cortex to inner medullar. Image was magnified at x4, Bar = 200 µm. **(c-f)** Tubule specific expression of PrdxV (Red) were assessed by tubule markers (Green), AQP1, THP, Calbindin D28K, and AQP2, using immunofluorescence. Nucleus was stained with DAPI. Image was magnified at x400.

Supplementary Figure. S2

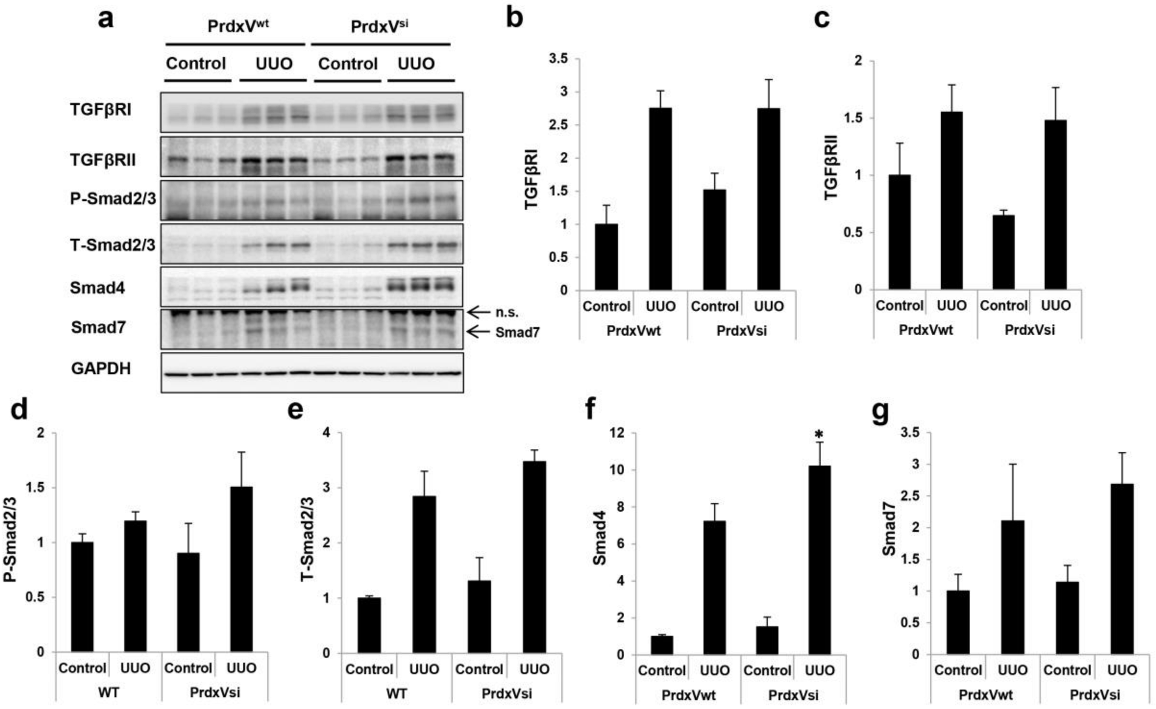

Canonical TGF-β signaling pathway in UUO-induced PrdxV^{si} mouse kidney. To verify the involvement of the canonical TGF-β signaling pathway in renal fibrosis aggravated by knockdown of PrdxV, the expression levels and activation levels of major TGF-β/Smad signaling molecules were assessed by western blotting **(a)**. GAPDH was used as an internal control. **(b-e)** Bar graphs show the mean target protein/GAPDH expression as measured by densitometry (**a**; TGFβRI, **c**; TGFβRI, **d**; Phospho-Smad2/3, **e**; Total-Smad2/3).

Uncropped western blotting data

Figure 2

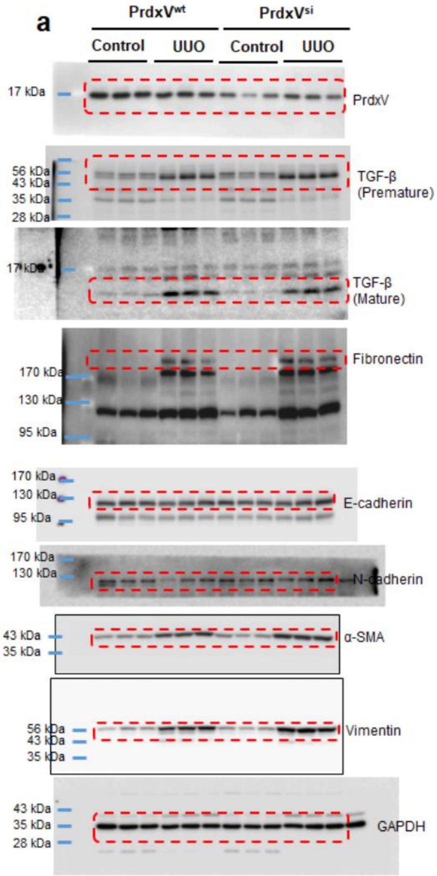

Figure 3

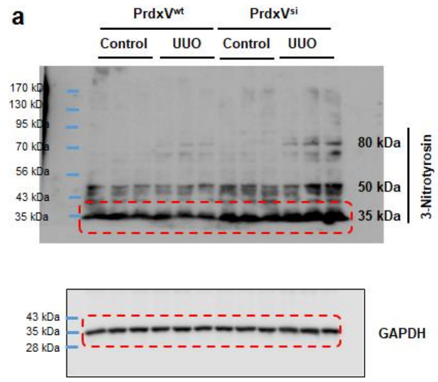

Figure 4

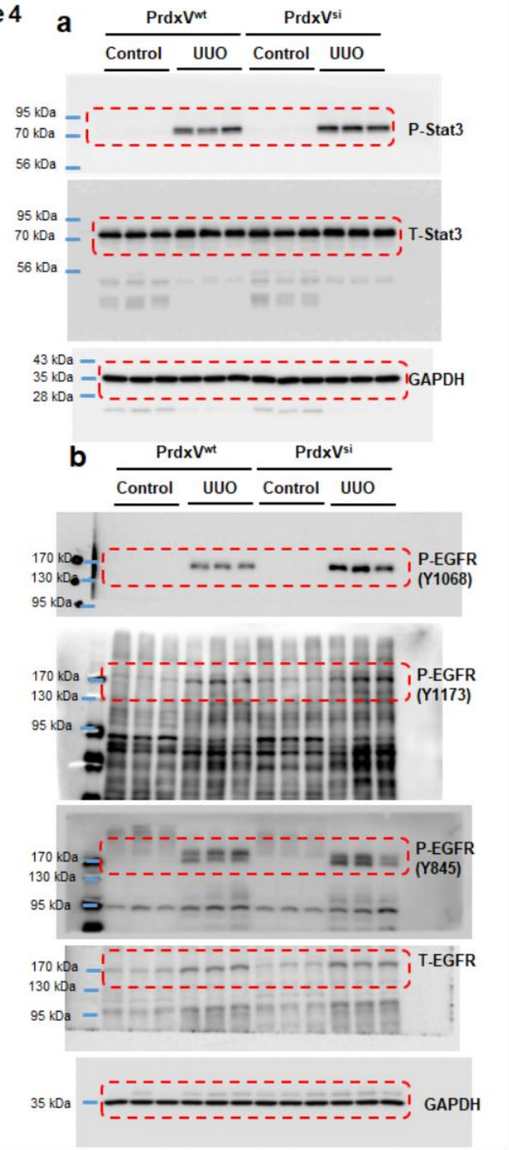

Figure 5

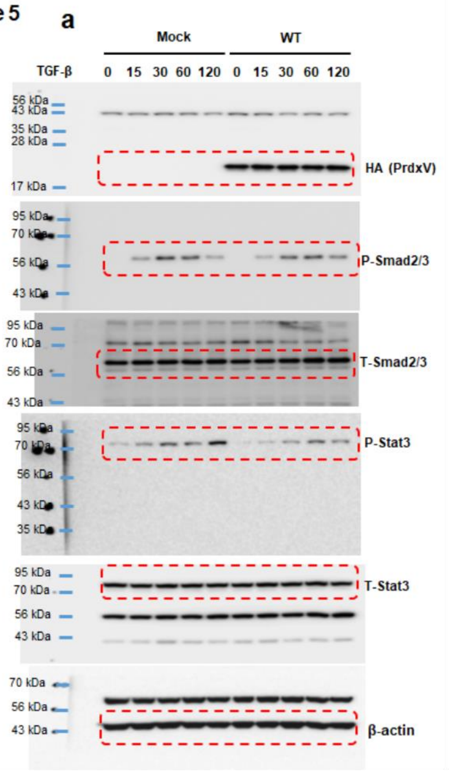

Figure 5

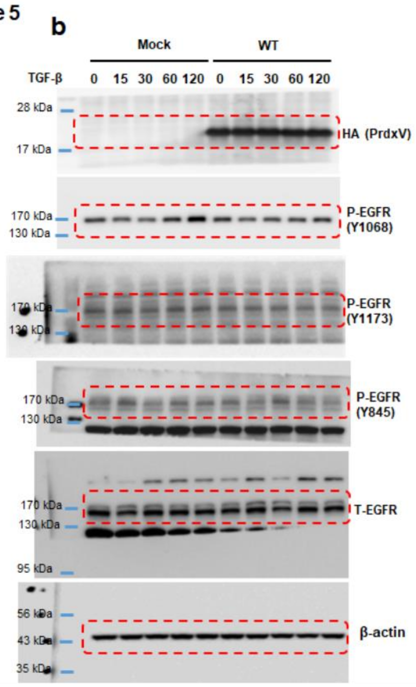

Figure 6

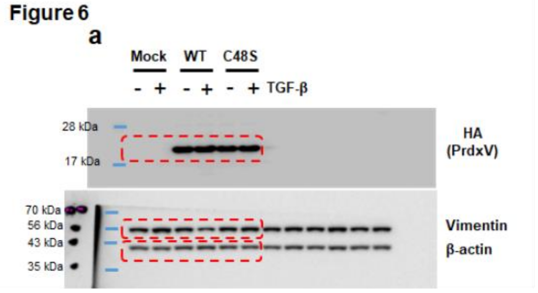

Figure 7

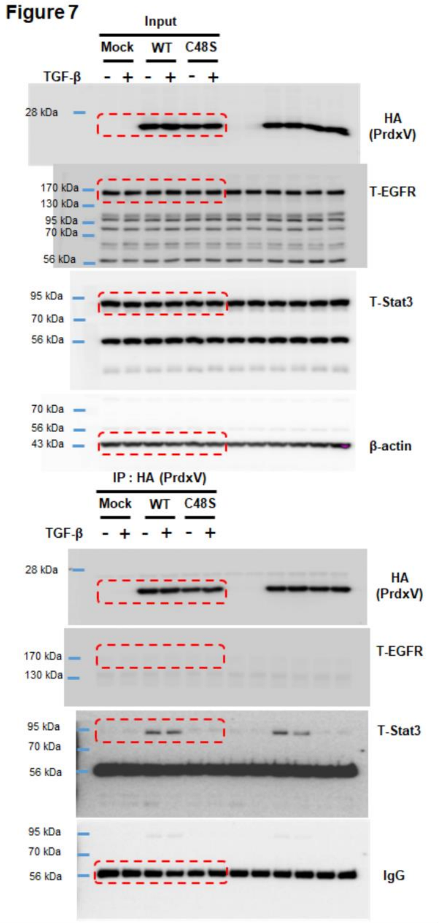

Supplementary Figure S1

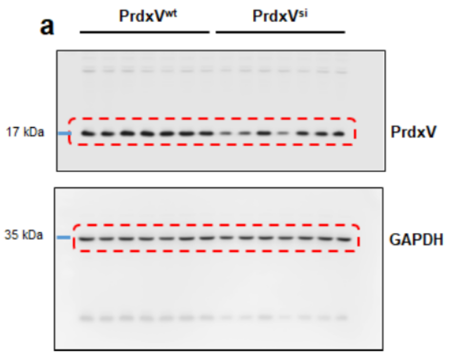

Supplementary Figure S2.

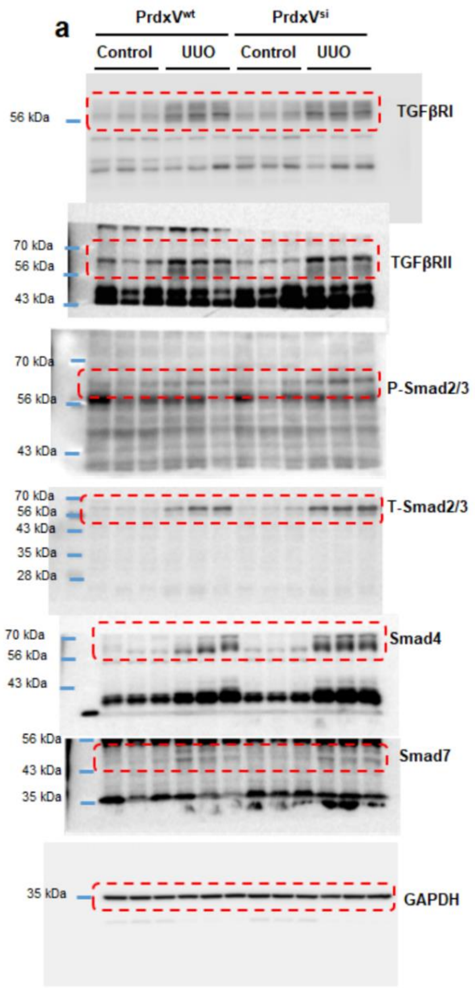

Supplement: Supplementary file 1 — Supplementary information [file 41598_2019_45347_MOESM1_ESM.pdf]
